# Visualizing Cyclic Adenosine Monophosphate in Cardiac Microdomains Involved in Ion Homeostasis

**DOI:** 10.3389/fphys.2019.01406

**Published:** 2019-11-26

**Authors:** Vladimir Dikolayev, Turlybek Tuganbekov, Viacheslav O. Nikolaev

**Affiliations:** ^1^Institute of Experimental Cardiovascular Research, University Medical Center Hamburg-Eppendorf, Hamburg, Germany; ^2^Department of Surgical Diseases, Astana Medical University, Nur-Sultan, Kazakhstan; ^3^German Center for Cardiovascular Research (DZHK), Hamburg, Germany

**Keywords:** cyclic adenosine monophosphate, microdomain, imaging, cardiomyocyte, Förster resonance energy transfer biosensor

## Abstract

3′,5′-Cyclic adenosine monophosphate (cAMP) is a key second messenger that regulates function of proteins involved in ion homeostasis and cardiac excitation-contraction coupling. Over the last decade, it has been increasingly appreciated that cAMP conveys its numerous effects by acting in discrete subcellular compartments or “microdomains.” In this mini review, we describe how such localized signals can be visualized in living cardiomyocytes to better understand cardiac physiology and disease. Special focus is made on targeted biosensors that can be used to resolve second messenger signals within nanometers of cardiac ion channels and transporters. Potential directions for future research and the translational importance of cAMP compartmentalization are discussed.

3′,5′-Cyclic adenosine monophosphate (cAMP) is a key second messenger that serves, among many ubiquitous functions, as a critical regulator of heart performance and cardiac disease. Stimulation of G_s_ protein-coupled receptors on heart muscle cells (cardiomyocytes), including most notably β-adrenergic receptors (β-ARs) stimulated by catecholamines, activates adenylyl cyclase (AC) that catalyzes the generation of cAMP. In turn, cAMP will directly activate protein kinase A (PKA), Epac nucleotide exchange factors for the Rap subfamily of RAS-like small GTPases, cAMP-dependent ion channels, and Popeye domain containing proteins. Classically, cAMP was considered a freely diffusible second messenger. However, in response to adrenergic signaling during physiological fight-or-flight responses, cAMP apparently acts within discrete subcellular microdomains to fine-tune ion channel and transporter activities controlling excitation-contraction coupling. In addition, upon chronic stimulation, compartmentalized cAMP will promote cardiac hypertrophy and remodeling (Fischmeister et al., 2006; Perera and Nikolaev, 2013; Bers et al., 2019). Such microdomains, referred to by some authors as “nanodomains” or “nanocompartments” due to their apparent nanometer scale dimension, are typically organized around scaffolding proteins that bind PKA called A-kinase anchoring proteins (AKAPs). cAMP-AKAP compartments have been described for L-type calcium channels (LTCCs) important for calcium influx, ryanodine receptors (RyRs) that mediate release of Ca^2+^ from intracellular stores, and phospholamban (PLN) that mediates calcium reuptake in diastole *via* its interaction with the sarcoplasmic/endoplasmic reticulum (SR) calcium ATPase 2a (SERCA2a) pump (Bers, 2002; Lompre et al., 2010; Froese and Nikolaev, 2015). Each of these proteins is part of a multimolecular complex containing anchored PKA molecules brought into close proximity to its substrates, specific isoforms of phosphodiesterase (PDE), which catalyze cAMP degradation to both terminate cAMP signaling and provide spatial restriction, as well as other kinases, phosphatases, and other signaling molecules that contribute to local cellular regulation. All these signaling enzymes act together in a highly localized fashion to confer specificity to the diverse physiological and pathophysiological responses triggered by the same second messenger cAMP (Buxton and Brunton, 1983; Mauban et al., 2009; Diviani et al., 2011; Scott et al., 2013). To better understand this type of regulation at the subcellular level and to modulate specific cAMP responses pharmacologically, one needs to gain much deeper insight into local microdomain-specific cAMP dynamics as currently possible only by state-of-the-art live cell imaging techniques.

## Cyclic Nucleotide Biosensors Enable Live Cell Imaging

The recently rapid development of Förster resonance energy transfer (FRET)-based biosensors has provided researchers with a versatile toolbox for real-time monitoring of cAMP in living cardiac cells (Sprenger and Nikolaev, 2013; Bers et al., 2019; Ghigo and Mika, 2019). cAMP biosensors usually have one of the following five types of design (see Table 1):

1. PKA holoenzyme is composed of two regulatory (R) and two catalytic (C) subunits. The first available FRET biosensor was composed of PKA holoenzyme with fluorescently labeled subunits to monitor its cAMP-dependent dissociation/re-association (Adams et al., 1991; Zaccolo and Pozzan, 2002).
2. Later, single-chain FRET biosensors were designed based on whole (Zhang et al., 2009; Herbst et al., 2011) or N-terminally truncated (DiPilato et al., 2004; Ponsioen et al., 2004) Epac1 or Epac2 sequence sandwiched between two fluorescent proteins such as enhanced cyan (CFP) and yellow (YFP) fluorescent proteins or their brighter and less pH sensitive analogues mTurquoise and circularly permuted Venus (Klarenbeek et al., 2015).
3. Fifteen years ago, we introduced a series of much more sensitive and compact CFP-YFP biosensors containing single cAMP binding domains from Epac1, Epac2, PKA, and HCN2 channels (Nikolaev et al., 2004, 2006; Norris et al., 2009). These and the aforementioned sensors have been modified to allow real time cAMP measurements in individual compartments as described below.
4. Recently, Zaccolo and colleagues have introduced a sensor called CUTie (cAMP Universal Tag for imaging experiments), which contains a single cAMP binding domain from PKA RIIβ subunit with YFP inserted into its loop 4–5 and CFP fused to the cAMP binding domain C-terminus (Surdo et al., 2017). This design allows generation of fusion proteins used as targeted biosensors with retained sensitivity and dynamic range. However, this strategy has so far been successful only for N-terminal fusions.
5. Very recently, non-FRET cAMP biosensors called Pink Flamindo and R-FlincA were developed based on the red fluorescent protein mApple by either inserting a single cAMP binding domain from Epac1 between amino acids 150 and 151 of mApple or by using a circularly permuted version of this fluorescent protein (cp146) inserted into the PKA regulatory RIα subunit, respectively (Harada et al., 2017; Ohta et al., 2018). No targeted versions of these biosensors have been described so far.

**Table 1:** Major types of design and examples of cAMP biosensors.

Design	Biosensor name	Dynamic range (%)	EC_50_ (μM)	References
1. Whole PKA heterotetramer chemically labeled with Fluorophores Fused to CFP and YFP	FlCRhR R-CFP;C-YFP	~20–30 ~ 20–30	0.09 0.5–0.9	Adams et al. (1991) Zaccolo and Pozzan (2002)
2. Whole-length or partially truncated Epac between CFP/YFP or their mutants	CFP-Epac1-YFP CFP-Epac2-YFP CFP-(δDEP, CD)-YFP (H30) ICUE1/2/3 Epac-S^H188^ Epac-S^H187^ (Epac-S^H188^ with Q270E mutation) (H187)	~15 ~10–20 ~50 ~20–30 ~100 ~160	~50 ~15 ~10–50 ~10 ~4	DiPilato et al. (2004); Ponsioen et al. (2004); Zhang et al. (2009); Herbst et al. (2011); Klarenbeek et al. (2015)
3. Single CNBD sandwiched between YFP and CFP	Epac1-camps Epac2-camps PKA-camps HCN2-camps Epac2-camps300 mlCNBD-FRET	~30 ~20 ~20 ~15 ~20 ~30–40	2.4 0.9 1.9 6 0.3 0.07	Nikolaev et al. (2004, 2006); Norris et al. (2009); Mukherjee et al. (2016)
4. Single CNBD with YFP in the loop 4–5, CFP at C-terminus	CUTie	~20	7.4	Surdo et al. (2017)
5. Non-FRET	Pink Flamindo2 R-FlincA	~400 ~600	7.2 0.3	Harada et al. (2017); Ohta et al. (2018)

*Dynamic range represents a maximally measured % change in FRET/fluorescence signal in cells or *in vitro*. EC_50_ shows the affinity of the sensor for cAMP*.

*CNDB, cyclic nucleotide binding domain*.

## Visualizing Local Cyclicamp in the Vicinity of Ion Channels and Transporters Using Targeted Biosensors

To monitor cAMP dynamics specifically in various microdomains, several targeted biosensors have been developed. If expressed at appropriate levels, FRET sensors that incorporate full-length PKA or Epac (1 and 2 above) can have a subcellular distribution comparable to that of the endogenous cAMP effector proteins, permitting visualization of varying cAMP levels in multiple PKA and Epac containing compartments across a cell. However, as individual cAMP compartments can be smaller than the resolution of light microscopy, that is, nanometers in diameter, the specific targeting of cAMP sensors to individual compartments is required if cAMP signaling within individual compartments is to be imaged independently of cAMP fluxes that may be quite distinct in neighboring intracellular compartments.

The N-terminal dimerization-docking domain of the PKA regulatory subunit confers AKAP binding. As a first approach to distinguish cAMP compartments, Zaccolo and colleagues fused these sequences derived from the two types of regulatory PKA subunits (RI and RII) to the N-terminus of Epac1-camps to monitor cAMP dynamics at the subcellular sites where endogenous type I and type II PKA are localized. This experimental strategy revealed that PKA type I and II participate in signaling compartments regulated by different PDE isoforms. Type I PKA is more prone to stimulation *via* prostaglandin and glucagon receptors, whereas type II PKA is predominantly regulated by β-ARs (Di Benedetto et al., 2008). These compartments seem to mediate distinct functional responses, although it is still not completely understood which particular set of substrates is preferentially phosphorylated by the different PKA holoenzymes. Type II PKA can phosphorylate LTCC, RyR, and PLN, a small protein which regulates the activity of the SERCA2a pump, whereas PKA type I substrates are still unknown or controversial (Di Benedetto et al., 2008). Despite providing powerful biosensors to monitor local cAMP responses, targeting sensors *via* cAMP effectors has limitations. For example, several AKAPs have been shown to bind each type of PKA regulatory subunits (Diviani et al., 2011), so that each of such sensors can be expected to be simultaneously present at multiple distinct locations in the cell, confounding signals from different compartments when imaged by standard resolution light microscopy. Also, expression of such biosensors would inevitably lead to displacement of endogenous PKA molecules from the microdomains of interest, so that PKA-dependent regulation of local cAMP, PDE, and ion channel activities might be at least partially altered. If possible, these limitations should be considered when designing new targeted biosensors.

**Figure 1:**
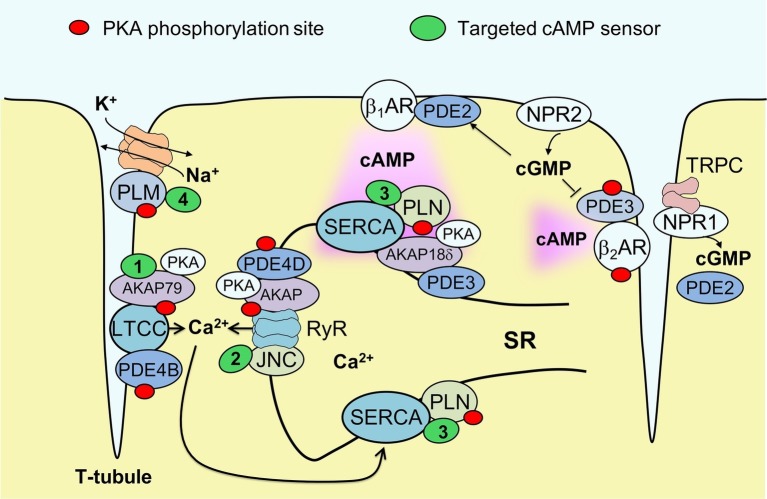
Targeted biosensors used to visualize cAMP in the vicinity of proteins regulating cardiac ion homeostasis. Excitation-contraction coupling in ventricular cardiomyocytes is regulated by the L-type calcium channels (LTCCs) located in membrane invaginations called T-tubules, as well as by sarcoplasmic calcium release (ryanodine receptors, RyR) and reuptake (SERCA pump). Each of these locales forms a microdomain regulated by a specific set of AKAPs and associated PDEs that controls local PKA phosphorylation (marked by red dots). SERCA activity is inhibited by the small regulatory protein phospholamban (PLN); this inhibition is reduced upon PKA mediated PLN phosphorylation. Sodium homeostasis is under tight control by the Na/K-ATPase (NKA) shown in orange. This pump is inhibited by phosholemman (PLM), a regulatory protein that is less active when PKA phosphorylated. cAMP produced after stimulation of β_1_-adrenergic receptors (βARs) diffuses across the cell, activating local pools of PKA in individual microdomains. cGMP can be produced by membrane guanylyl cyclases represented by natriuretic peptide receptors (NPRs) and can either activate PDE2 or inhibit PDE3. NPR1 is confined to T-tubules where it directly interacts with TRPC channels and produces a local pool of cGMP controlled by PDE2. To measure cAMP directly in the vicinities of each ion channel or transporter, targeted FRET biosensors (green) have been developed, such as AKAP79-CUTie (1, for LTCC), Epac1-JNC (2, for RyR), Epac1-PLN or AKAP18δ-CUTie (3, for SERCA) and PLM-Epac1 (4, for the NKA microdomain). See text for details. JNC – junctin, regulatory protein which forms a stable complex with RyR.

To gain further insight into microdomains around calcium handling proteins, our group has developed new versions of the cAMP biosensor Epac1-camps that are localized either to caveolin-rich plasma membrane (Perera et al., 2015) or to major SR membrane proteins SERCA2a and RyR, by fusing the sensor to PLN (Sprenger et al., 2015) or junctin (Berisha et al., 2019), respectively (see Figure 1). These sensors have been expressed in ventricular myocytes of transgenic mice to allow the imaging of cAMP in both normal and disease states. In experiments using myocytes isolated from mice subjected to pressure overload to induce cardiac hypertrophy and early heart failure, these biosensors uncovered a disease driven intracellular redistribution of several PDE families. For example, PDE2 was shown to switch locations between β_1_- and β_2_-AR-associated plasma membrane microdomains (Perera et al., 2015), while PDE3A switched isoforms from A2 and A1 and relocated from the sarcolemma to the SR (Perera et al., 2015; Berisha et al., 2019). This type of PDE redistribution had a functional impact on myocyte contractility, enhancing β_1_-AR mediated inotropic increases in contractile force, especially when natriuretic peptide receptors were co-stimulated (Perera et al., 2015). However, microdomain-specific changes in PDE localization apparently occur before a measurable decrease of β_1_-AR and PDE3/4 expression at the protein level associated with the onset of ventricular dilation resulting in heart failure with reduced ejection fraction (HFrEF). Therefore, PDE redistribution might contribute to the relative preservation of systolic function during early pathological cardiac remodeling induced by pressure overload, without providing protection from fatal arrhythmia. Arrhythmias in heart failure are also associated with sodium overload. We recently studied healthy and HFrEF rat ventricular myocytes using another newly developed biosensor fused to phospholemman to enable cAMP monitoring in the vicinity of the major cardiac sodium pump, the Na/K-ATPase (see Figure 1). We found that the phospholemman microdomain is normally regulated by β_2_-AR/cAMP and PDE3, while induction of HFrEF by myocardial infarction resulted in a pronounced exchange of PDE3 with PDE2 in this microdomain (Bastug-Özel et al., 2019). The power of targeted biosensors is illustrated by the fact that most of these early pathological alterations in cAMP compartmentation cannot be detected using non-localized, cytosolic sensors or traditional biochemical techniques. Collectively, these studies suggest that each individual microdomain regulating ion homeostasis has a unique local cAMP regulation profile that might change in disease and alter cellular function. An important challenge will be to develop therapeutic strategies to target specifically these microdomains in disease, selectively promoting those cAMP-dependent processes that preserve cardiac function.

Targeted cAMP biosensors have also provided insight into the variation in cAMP signaling induced by stimulation of different cell membrane receptors. For example, in HEK293 cells, cAMP levels monitored by variants of the H30 biosensor (see Table 1) targeted to the cytosol, plasma membrane, and nucleus were found after prostaglandin receptor stimulation to be relatively high at the plasma membrane, significantly lower in the bulk cytosol and again much higher in the nucleus (Terrin et al., 2006). Likewise, β_1_-AR stimulation in adult mouse ventricular cardiomyocytes led to higher cAMP levels detected at SERCA2a using Epac1-PLN sensor as compared to bulk cytosol (Sprenger et al., 2015). Also, cAMP increase measured in the vicinity of AKAP18δ (associated with SERCA2a) and AKAP79 (associated with LTCC) imaged using targeted versions of CUTie were higher than in the bulk cytosol (Surdo et al., 2017). Interestingly, this was not the case for a myofilament-associated microdomain imaged using CUTie fused to troponin I, which showed relatively low cAMP levels at submaximal β-adrenergic stimulation. This spatial heterogeneity was further accentuated under pathological conditions (Surdo et al., 2017). These studies suggest the existence of a privileged receptor/microdomain or receptor/calcium handling protein “communication.” Mechanistically, this process could be mediated by the concerted action of different PDEs in different compartments (Sprenger et al., 2015). Alternatively, it has been proposed that active G protein-coupled receptors are present on intracellular membranes such as the nuclear envelope or trans-Golgi network where β-ARs, for example, can be activated by externally applied ligands that gain entry *via* organic cation transporters and activate cAMP production deep inside the cell (Irannejad et al., 2017). However, this possibility is not yet extensively studied in cardiac cells.

One way to visualize receptor/microdomain communication is to combine targeted biosensors with scanning ion conductance microscopy (SICM). SICM is a non-optical imaging technique which utilizes an electrolyte-filled glass nanopipette as a scanning probe for noncontact visualization of live cell membrane morphology (Korchev et al., 1997). The current flowing through the SICM pipette tip is decreased whenever the tip approaches the cell membrane. By clamping this drop in current at a fixed value, the distance between pipette tip and the membrane can be kept constant, so that scanning the membrane in the X-Y direction can resolve the morphological profile of the membrane with nanometer resolution. SICM as a multimodal imaging technique is useful not only for single cells but also for intact tissue structures (Miragoli et al., 2011). By combining SICM with FRET recordings of cAMP performed during local receptor stimulation *via* agonist delivery through the scanning pipette, we found that β_1_-ARs were diffusely present on the surface and T-tubules of the myocyte plasma membrane, whereas β_2_-AR was found exclusively in the T-tubules of healthy rat and mouse cardiomyocytes (Nikolaev et al., 2010). Likewise, natriuretic peptide receptors NPR2 and NPR1 show a similar differential distribution pattern on the myocyte plasma membrane resulting in compartmentation of cyclic guanosine monophosphate (cGMP) signaling (Subramanian et al., 2018). Strict confinement of β_2_-AR and NPR1 stimulated second messenger signals to T-tubular membrane are mediated by local PDE activities (mostly PDE4 and PDE2, respectively) and are likely to change in disease due to a redistribution of receptors and PDEs, as shown for β_2_-AR and PDE2-4 (Perera et al., 2015; Sprenger et al., 2015). In the future, it would be exciting to combine SICM with targeted cAMP biosensors and with simultaneous ion channel recordings (Sanchez-Alonso et al., 2016) to directly visualize how receptors stimulated at different membrane locations regulate ion channel currents in microdomains. In this case, use of SICM allows activation of spatially resolved membrane structures, such as single T-tubules, with nanometer precision to activate a discrete pool of receptors, without affecting them at other membrane locations. When combined with localized FRET biosensor readouts in certain microdomains inside the cell, this approach provides a possibility to dissect the aforementioned receptor/microdomain communication more directly. It will be exciting to explore whether the same receptor generates different cAMP responses at different subcellular locations and if they are affected by cardiac disease.

## Future Perspectives

The aforementioned studies have provided an introduction into the microdomains associated with proteins regulating cardiac ion homeostasis. Beyond any doubt, this exciting field of research is still at its very beginning and much deeper insights will follow with time. We propose that future research should include the following topics for investigation:

1. Other differentially targeted biosensors with improved sensitivity and signal-to-noise should be developed and applied to imaging cAMP at ion channels and transporters involved in cardiac function and disease. For excitation-contraction coupling, targets of interest include the Nav1.5 sodium channel, the sodium-calcium exchanger and the potassium channel KCNQ1. All of them can be PKA phosphorylated, and notably KCNQ1 binds an AKAP called Yotiao, whose mutation results in long QT syndrome (Chen et al., 2007). For hypertrophy, an interesting microdomain is likely to be the vicinity of transient receptor potential C (TRPC) channels which is directly associated with NPR1 (Klaiber et al., 2011) (see Figure 1). When designing targeted biosensors, it is important to make sure that they do not perturb the composition and function of cAMP microdomains, for example, due to displacement of endogenous proteins or by altering microdomain regulation. For each newly developed sensor and cells expressing it, a detailed analysis of biochemical and functional properties pertaining to the microdomain of interest should be performed.
2. *In vivo* mouse models should be developed to dissect the individual contributions of specific PDE, AC, and AKAP families and subfamilies to derive insight into local cAMP signal regulation within individual microdomains. For example, at least three different PDE4 subfamilies (A, B and D) and two PDE3 subfamilies (A and B) have been implicated in local cAMP regulation in the cardiac myocyte (Conti and Beavo, 2007; Movsesian et al., 2018). PDE4B is functionally associated with LTCC and PDE4D with RyR (Lehnart et al., 2005; Leroy et al., 2011). Revisiting these studies using targeted biosensors in combination with PDE subfamily specific knockout mice should provide direct microdomain-specific readouts for individual locally acting PDEs and better link them to cardiac function and disease. Most PDE and AKAP global knockout mouse models do not allow the analysis of specific isoform contributions, for example, those of PDE4D3, PDE2A2 or AKAP18δ. There are also no specific isoform-selective pharmacological inhibitors available which could be used for this purpose. Rapid development of CRISPR/Cas9 gene editing technology should be able to overcome this limitation, providing new interesting mouse models or even specific deletions of individual isoforms in primary isolated cardiomyocytes *ex vivo*. Alternatively, for the PDE4 family in which localization is conferred by alternatively-spliced N-terminal sequences, expression of anchoring disruptor peptides may be particularly useful.
3. The aforementioned small and large animal disease models can be combined with gene therapy approaches aimed at overexpression of microdomain protein components which are downregulated in heart failure such as some PDEs, as it has been done in the pioneering work with SERCA2a (Jessup et al., 2011). Restoration of the expression of individual PDEs may restore normal local cAMP dynamics in functionally relevant microdomains and ameliorate disease-associated cardiac dysfunction. Conversely, disruption of PDE-microdomain interactions using specific peptides can be instrumental in augmenting cardioprotective cyclic nucleotide pools, as elegantly shown by Baillie and colleagues for the PDE4D3/Hps20 interaction (Sin et al., 2011). A similar approach is now under evaluation for the SERCA2a/PDE3A complex. Notably, this latter strategy has been deployed outside the cardiovascular system. For example, expression of the 4D3 N-terminal peptide has been used to disrupt mAKAPα-PDE4D3complexes in the retinal ganglion cell to confer neuroprotection (Boczek et al., 2019).
4. It will be important to transit from imaging cardiac myocytes *in vitro*, whether following culture or immediately *ex vivo* after isolation from mice, to imaging myocytes *in vivo* in the living heart. Recently, developed cAMP imaging in isolated Landgendorff perfused hearts that can be combined with electrophysiological recordings (Jungen et al., 2017) or even in open chest *in vivo* models (Tallini et al., 2006; Frankenreiter et al., 2017) are helping us achieve imaging in a physiological context. Interestingly, cAMP responses and the biochemical composition of signalosomes comprising individual compartments can vary depending upon of the anatomical origin of the myocytes, with differences recognized between the atria and base and apex of the ventricles (Wright et al., 2018). These differences should be more carefully considered in the future studies.
5. Last but not least, it will be important to determine whether cyclic nucleotide compartmentation is conserved among mammalian species, including most importantly between rodents and humans. Most research in this field has been performed in rats and mice, given the difficulty in acquiring adult human cardiac myocytes, with the notable exception of a few studies taking advantage of atrial tissue acquired from patients with atrial fibrillation (Molina et al., 2012; Glukhov et al., 2015). Since structure of atrial and ventricular cells and their biochemical makeup including expression of receptors and PDEs are different, additional studies have to be undertaken to better compare mechanisms of cAMP compartmentation in atrial vs. ventricular myocytes. Human studies are of obvious translational importance, and the derivation of cardiac myocytes from human induced pluripotent stem cells (hiPSC-CMs) should ultimately facilitate this research. A current major limitation with using hiPSC-CMs is that current procedures for “maturing” the myocytes yield cells similar to fetal or neonatal myocytes that have significant differences in ultrastructure and excitation-contraction coupling from adult myocytes *in vivo*, an issue of obvious relevance to the study of compartmentalized signaling. Nevertheless, using a cytosolic cAMP biosensor, clear differences in β-adrenergic sensitivity and PDE function were detected using hiPSC-CM for patients with Takotsubo cardiomyopathy (Borchert et al., 2017). This landmark study should be extended using localized FRET biosensors to better understand microdomain alterations in this and other cardiac diseases. If these alterations are comparable to those happening in adult human cells, hiPSC-CMs could be useful to screening drugs for new cardiovascular therapeutics targeting cyclic nucleotide compartmentation, much as hiPSC-CMs have proven useful for diagnosis and analysis of inherited ion channel diseases such as Long QT syndrome (Sinnecker et al., 2013).

## Author Contributions

VD, TT, and VN discussed the concept, wrote, and edited the manuscript.

### Conflict of Interest

The authors declare that the research was conducted in the absence of any commercial or financial relationships that could be construed as a potential conflict of interest.
